# Long-term efficacy and renal safety of SGLT2 inhibitors in patients with heart failure and advanced chronic kidney disease (stage 4): a propensity score-matched retrospective cohort study

**DOI:** 10.3389/fcvm.2026.1849205

**Published:** 2026-06-17

**Authors:** Weihua Peng, Xiaoke Shang, Changdong Zhang, Mei Liu

**Affiliations:** 1 Department of Cardiology, Fugou Huimin Traditional Chinese Medicine Hospital, Zhoukou, Henan, China; 2 Department of Cardiovascular Surgery, Union Hospital, Tongji Medical College, Huazhong University of Science and Technology, Wuhan, Hubei, China; 3 Cardiac Laboratory, Department of Cardiovascular Surgery, Union Hospital, Tongji Medical College, Huazhong University of Science and Technology, Wuhan, Hubei, China; 4 Department of Nutrition, Wuhan No.1 Hospital, Wuhan, Hubei, China

**Keywords:** cardiorenal syndrome, chronic kidney disease, heart failure, propensity score matching, sodium-glucose cotransporter 2 inhibitors

## Abstract

**Objective:**

Sodium-glucose cotransporter 2 inhibitors (SGLT2i) are a cornerstone of heart failure (HF) therapy; however, landmark trials systematically excluded patients with severe renal impairment. Consequently, prescribing SGLT2i for HF patients with advanced chronic kidney disease (CKD stage 4) remains a clinical “blind spot” due to fears of acute kidney injury. This study aimed to evaluate the long-term efficacy and renal safety of SGLT2i in this vulnerable, real-world population.

**Methods:**

This single-center, retrospective observational cohort study evaluated HF patients with a baseline estimated glomerular filtration rate (eGFR) of 15–29 mL/min/1.73 m^2^ treated between January 2021 and December 2024. To minimize confounding by indication, a rigorous 1:1 propensity score matching (PSM) was performed. The primary efficacy outcome was a composite of heart failure hospitalization (HHF) or cardiovascular death. The primary safety outcome was major adverse kidney events (MAKE), defined as a sustained ≥40% eGFR decline, initiation of dialysis, or renal death. Long-term eGFR slopes were analyzed using linear mixed-effects models.

**Results:**

Following PSM, the final analysis cohort comprised 320 balanced patients (160 in the SGLT2i group and 160 in the control group), with a median baseline eGFR of 22.4 mL/min/1.73 m^2^. Over a median follow-up of 2.1 years, the SGLT2i group demonstrated a 38% relative risk reduction in the primary efficacy composite outcome compared to controls (30.6% vs. 45.6%; adjusted HR 0.62, 95% CI 0.44–0.87, *P* = 0.005). The incidence of the primary safety outcome (MAKE) was statistically comparable between the two groups (14.4% vs. 16.9%; adjusted HR 0.86, 95% CI 0.52–1.43, *P* = 0.550), with no significant excess in severe acute kidney injury or hyperkalemia. Following an initial early “dip” in eGFR (−2.1 mL/min/1.73 m^2^), SGLT2i utilization was associated with a significantly shallower annualized eGFR decline trajectory compared to standard care (−1.2 vs. −3.5 mL/min/1.73 m^2^ per year, *P* < 0.001).

**Conclusions:**

In high-risk, real-world HF patients complicated by advanced CKD (Stage 4), SGLT2i therapy appears to be associated with substantial cardiovascular benefits and may attenuate long-term renal function decline. These findings suggest that severe renal impairment should not instinctively preclude the utilization of this life-saving therapy.

## Introduction

Heart failure (HF) and chronic kidney disease (CKD) frequently coexist, creating a complex pathophysiological spiral often referred to as cardiorenal syndrome. Epidemiological data suggest that up to 50% of patients with HF present with concomitant renal impairment, which is unequivocally associated with a disproportionately high rate of cardiovascular mortality and recurrent hospitalizations (1). Over the past decade, sodium-glucose cotransporter 2 inhibitors (SGLT2i) have emerged as a cornerstone of HF management, transforming the treatment landscape (2). Large-scale randomized controlled trials (RCTs) such as DAPA-HF, EMPEROR-Reduced, and DELIVER have robustly demonstrated that SGLT2i significantly reduce the risk of cardiovascular death and hospitalization for HF across the spectrum of left ventricular ejection fractions (LVEF), establishing them as a mandatory pillar of the “fantastic four” foundation therapy in international guidelines (3–5).

Despite these monumental advancements, a critical knowledge gap remains. To ensure patient safety, the landmark RCTs defining the guideline status of SGLT2 inhibitors rigorously excluded patients with severe renal impairment—specifically those with an estimated glomerular filtration rate (eGFR) less than 25 or 30 mL/min/1.73 m^2^, a common threshold in foundational trials (6, 7). Consequently, patients with advanced CKD (Stage 4), representing an extremely vulnerable and ultra-high-risk population, have been systematically underrepresented in high-quality evidence generation, as their inclusion in pivotal trials has been limited (8, 9). While recent renal-focused trials like DAPA-CKD and EMPA-KIDNEY have provided robust safety data down to eGFR levels of 25 and 20 mL/min/1.73 m^2^, evidence specifically detailing heart failure outcomes in these advanced CKD patients remains relatively scarce. In real-world clinical practice, physicians frequently encounter this precise demographic. Yet, a pervasive “prescription hesitation” persists due to fears of inducing acute kidney injury (AKI) or precipitating an acute eGFR dip, rendering this frail population a distinct “blind spot” in evidence-based medicine (10, 11).

Given the strict exclusion criteria of apex RCTs, there is a pressing need to bridge this evidence gap using high-granularity, real-world data. Therefore, the objective of this study was to systematically evaluate the long-term cardiovascular efficacy and renal safety of SGLT2i compared to standard guideline-directed medical therapy (GDMT) in a highly vulnerable cohort of HF patients complicated by advanced CKD (Stage 4). By employing rigorous propensity score matching (PSM) to adjust for confounding biases inherent in observational research, we aimed to explore whether the prognostic benefits associated with SGLT2i persist in severe renal impairment, and to evaluate the corresponding renal safety profile.

## Methods

### Study design and ethics

This was a single-center, retrospective, observational cohort study conducted at a large tertiary cardiovascular center. The study protocol fully adhered to the ethical principles outlined in the Declaration of Helsinki (12) and was approved by the Institutional Review Board (IRB) of the study center. Given the retrospective nature of the analysis utilizing de-identified electronic medical records (EMR), the requirement for informed consent was waived by the ethics committee. This study was reported in adherence to the Strengthening the Reporting of Observational Studies in Epidemiology (STROBE) guidelines (13).

### Study population

**Figure 1:**
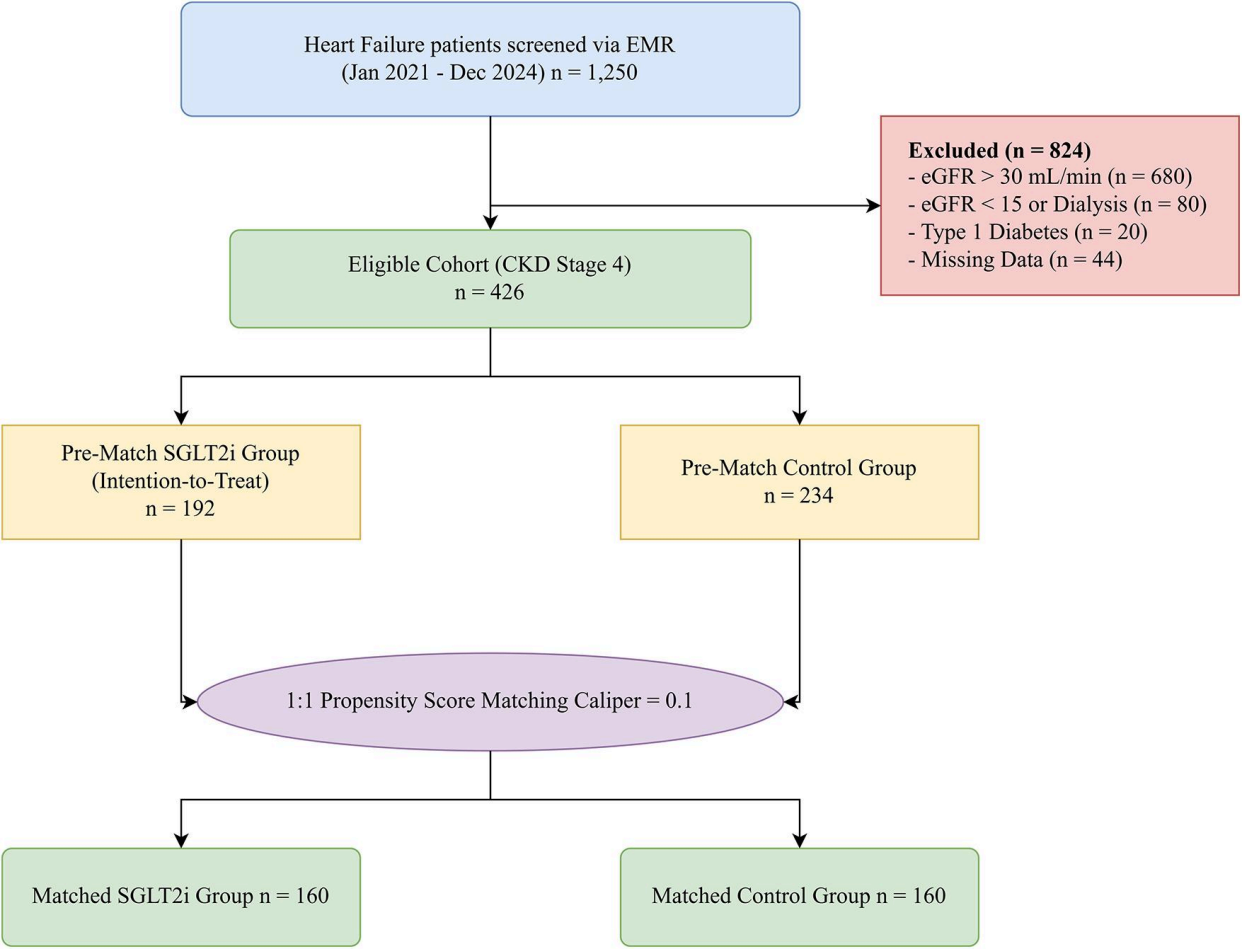
Study flowchart. The flowchart illustrates the patient selection process, exclusion criteria, and the generation of the final analytical cohort through 1:1 propensity score matching.

We systematically screened EMR data for patients who were either hospitalized for worsening heart failure (HF) or evaluated in the dedicated outpatient HF clinic between January 1, 2021, and December 31, 2024. The inclusion criteria were defined as follows: (1) principal diagnosis of heart failure; (2) baseline estimated glomerular filtration rate (eGFR) strictly ranging between 15 and 29 mL/min/1.73 m^2^ according to the Kidney Disease: Improving Global Outcomes (KDIGO) classification (14); and (3) age ≥18 years. We excluded patients based on the following criteria: (1) documented type 1 diabetes mellitus; (2) current receipt of renal replacement therapy (RRT); (3) terminal malignancies with a life expectancy of less than 6 months; and (4) missingness (>10%) in baseline covariate or follow-up data (Figure 1 details the patient flow).

### Exposure and cohort definition

The index date was defined as the date of hospital discharge or the initial outpatient clinic visit. Patients who fulfilled the eligibility criteria were divided into two cohorts based on their EMR prescription records at the index date following a strict Intention-to-Treat (ITT) principle to emulate a target trial and avoid immortal time bias (15). The SGLT2i group consisted of patients who were prescribed either dapagliflozin (10 mg daily) or empagliflozin (10 mg daily). The Control group comprised patients receiving conventional, guideline-directed medical therapy for HF, with no documented prescription of any SGLT2i in the 6 months preceding and including the index date.

### Follow-up and endpoints

Follow-up data were comprehensively gathered through EMR chart reviews, cross-linkage with the National Death Registry system, and structured telephone interviews. The end of the follow-up period was uniformly set to December 31, 2025, ensuring a minimum theoretical follow-up of 12 months for the latest enrolled subjects. Follow-up commenced on the index date. In time-to-event analyses, patients were censored at the date of the last clinical contact, death (for non-mortality outcomes), or if control patients were subsequently prescribed an SGLT2i during the follow-up period (as-treated censoring).

The primary efficacy outcome was a composite of the first hospitalization for heart failure (HHF) or cardiovascular death. The primary safety outcome was major adverse kidney events (MAKE), defined as a sustained ≥40% decline in eGFR from baseline, initiation of chronic RRT, or death attributable to renal causes, consistent with definitions used in recent landmark trials (16). Secondary outcomes included all-cause mortality, the long-term trajectory of eGFR decline (eGFR slope), the incidence of acute kidney injury (AKI) defined by the KDIGO criteria (17), and the occurrence of severe hyperkalemia (serum potassium >6.0 mmol/L). Severe hyperkalemia was included to evaluate whether SGLT2i could mitigate the hyperkalemia risk frequently associated with the concurrent use of Mineralocorticoid Receptor Antagonists (MRAs) in this severe CKD cohort.

### Statistical analysis

Baseline characteristics are presented as means with standard deviations (SD) or medians with interquartile ranges (IQR) for continuous variables, and as frequencies with percentages for categorical variables. Variables with less than 10% missing data were imputed utilizing the multiple imputation by chained equations (MICE) methodology (18). To mitigate the profound confounding by indication inherent to observational studies, particularly regarding the prescription of SGLT2i in severe CKD, we utilized rigorous Propensity Score Matching (PSM). Propensity scores were generated using a multivariable logistic regression model incorporating comprehensive covariates: patient demographics, vital signs (Systolic Blood Pressure, BMI), baseline comorbidities (including HF etiology and CKD etiology), echocardiographic parameters (LVEF), core laboratory markers (NT-proBNP, baseline eGFR), and concurrent cardioprotective medications (ACEI/ARB/ARNI, beta-blockers, MRA). We performed a 1:1 nearest-neighbor matching without replacement, enforcing a strict caliper of 0.1 standard deviations of the logit of the propensity score (19). The balance of covariates post-matching was assessed using the Standardized Mean Difference (SMD), with a threshold of <0.1 indicating excellent balance.

Time-to-event outcomes were visualized using Kaplan–Meier survival curves, and differences between groups were evaluated via the Log-rank test. Cox proportional hazards regression models were utilized to compute hazard ratios (HR) and their 95% confidence intervals (CI) for both the primary and secondary clinical endpoints. The longitudinal trajectory of eGFR over the 24-month follow-up was analyzed using Linear Mixed-Effects Models to account for repeated measures within subjects. Subgroup analyses for the primary efficacy endpoint were conducted and visualized using forest plots, assessing interactions across strata of age, LVEF, and diabetes status. A two-sided *P*-value <0.05 was considered statistically significant. All analyses were conducted using R software, version 4.3.1 (R Foundation for Statistical Computing, Vienna, Austria).

## Results

### Study population and baseline characteristics post-PSM

**Figure 2:**
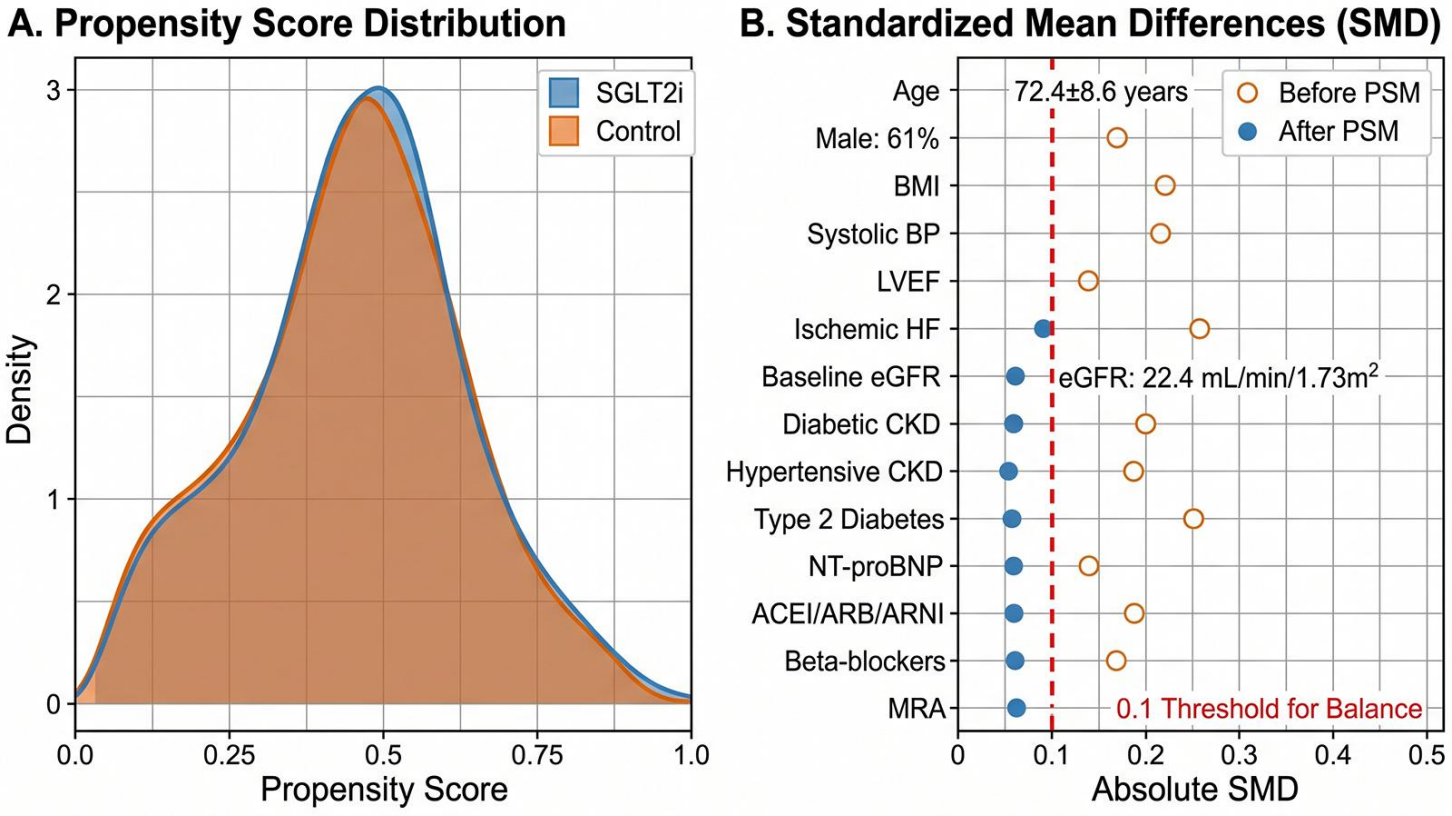
Assessment of propensity score matching quality. **(A)** Density plot demonstrating the distribution of propensity scores before and after matching. **(B)** Love plot displaying the absolute standardized mean differences (SMD) of covariates; post-matching SMDs strictly converge within the 0.1 equivalence threshold.

A total of 1,250 patients meeting the initial inclusion criteria were identified between January 2021 and December 2024. Following the application of exclusion criteria, 426 eligible patients remained. Given the non-randomized allocation of treatment, significant baseline imbalances were observed. Following 1:1 PSM with a stringent 0.1 caliper, the final analysis cohort comprised 320 patients, evenly distributed with 160 patients in the SGLT2i group and 160 in the control group (Figure 1). As summarized in Table 1, post-matching baseline characteristics were balanced. The mean age was 72.3 ± 8.7 years, 61.3% were male, and the median baseline eGFR was severely impaired at 22.4 mL/min/1.73 m^2^ (IQR 18.5–26.2). Furthermore, Figures 2A,B graphically confirm the adequacy of the match, demonstrating complete overlap in propensity score density and SMD values <0.1 for all included covariates.

**Table 1:** Baseline characteristics before and after PSM.

Characteristics	Before PSM (*N* = 426)			After PSM (*N* = 320)		
	SGLT2i (*n* = 192)	Control (*n* = 234)	SMD	SGLT2i (*n* = 160)	Control (*n* = 160)	SMD
Age, years (mean ± SD)	70.0 ± 8.2	74.3 ± 9.4	0.49	72.3 ± 8.7	72.5 ± 8.9	0.02
Male gender, *n* (%)	125 (65.1)	128 (54.7)	0.21	98 (61.3)	97 (60.6)	0.01
BMI, kg/m^2^ (mean ± SD)	26.4 ± 4.1	25.0 ± 3.9	0.35	25.7 ± 4.0	25.6 ± 3.8	0.03
Systolic BP, mmHg (mean ± SD)	127 ± 15	121 ± 17	0.35	125 ± 15	125 ± 16	0.00
LVEF, % (median, IQR)	39 (31–46)	41 (32–50)	0.28	40 (31–48)	41 (32–49)	0.04
HF etiology: ischemic, *n* (%)	99 (51.6)	144 (61.5)	0.20	85 (53.1)	86 (53.8)	0.01
Baseline eGFR, mL/min/1.73 m^2^ (median)	24.1	21.5	0.39	22.4	22.1	0.03
CKD etiology: diabetic nephropathy, *n* (%)	91 (47.4)	123 (52.6)	0.10	77 (48.1)	78 (48.8)	0.01
CKD etiology: hypertensive, *n* (%)	64 (33.3)	73 (31.2)	0.05	51 (31.9)	53 (33.1)	0.03
Type 2 diabetes, *n* (%)	94 (49.0)	133 (56.8)	0.16	80 (50.0)	82 (51.3)	0.03
NT-proBNP, pg/mL (median)	3,500	4,150	0.26	3,800	3,900	0.03
ACEI/ARB/ARNI use, *n* (%)	151 (78.6)	155 (66.2)	0.28	118 (73.8)	120 (75.0)	0.03
Beta-blockers use, *n* (%)	163 (84.9)	181 (77.4)	0.19	133 (83.1)	136 (85.0)	0.05
MRA use, *n* (%)	99 (51.6)	96 (41.0)	0.22	73 (45.6)	75 (46.9)	0.03

### Primary and secondary efficacy outcomes

**Figure 3:**
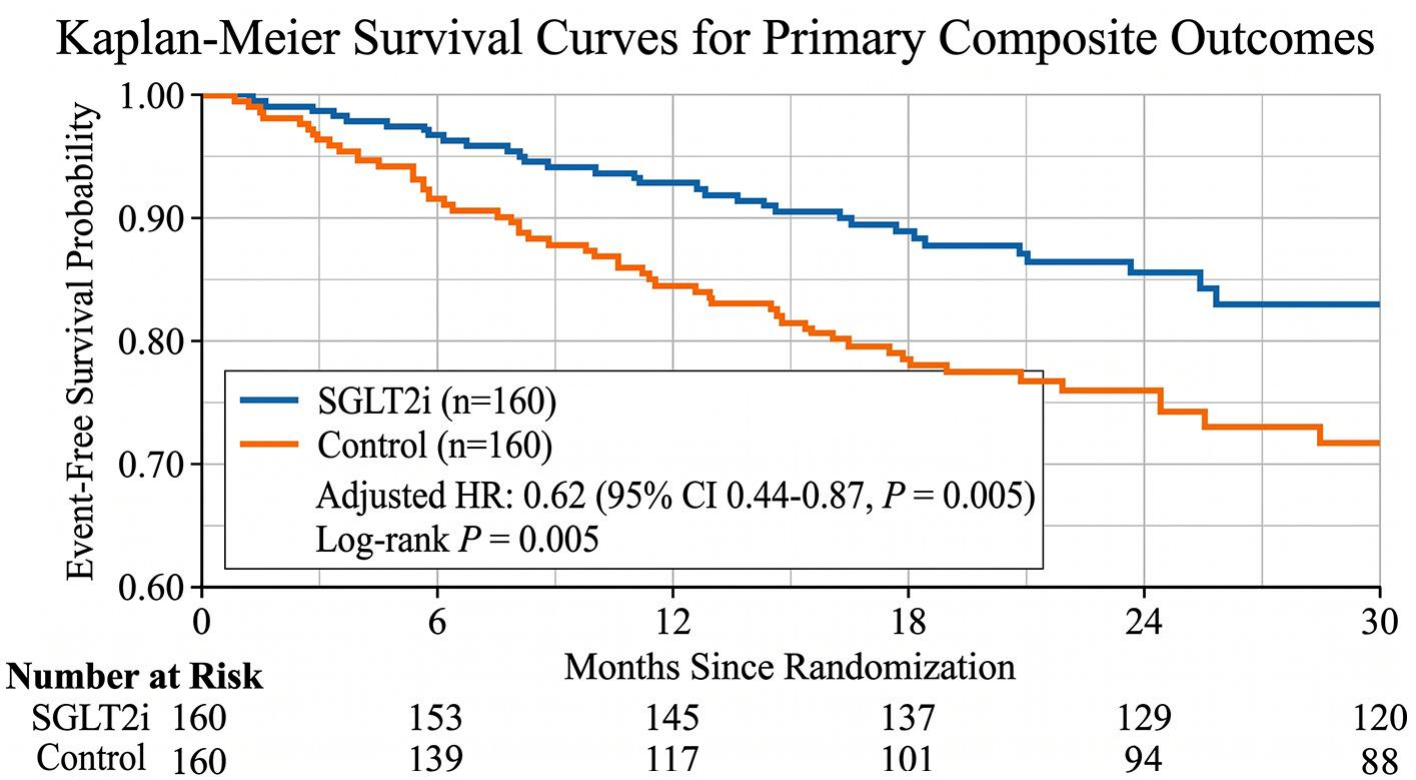
Kaplan–Meier Survival curves for primary outcomes. Event-free survival for the primary composite efficacy endpoint (heart failure hospitalization or cardiovascular death) stratified by SGLT2i vs. Control group. The number at risk is provided at 6-month intervals.

**Figure 4:**
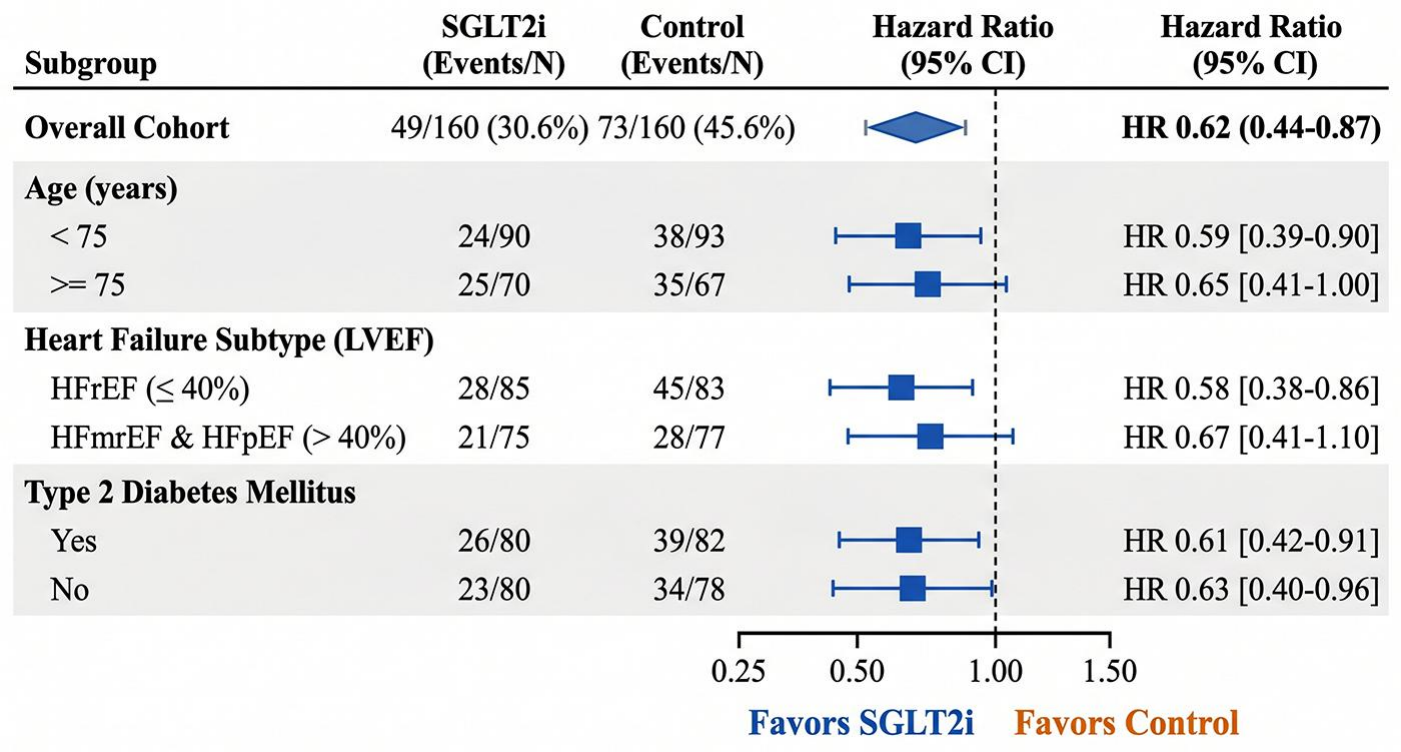
Forest plot of subgroup analyses. Subgroup analyses for the primary efficacy endpoint assessing the interaction of SGLT2i treatment effects across diverse clinical demographic strata.

Over a median follow-up of 2.1 years (IQR 1.4–3.2), the primary composite efficacy outcome (HHF or CV death) occurred in 49 patients (30.6%) in the SGLT2i group compared to 73 patients (45.6%) in the control group. Kaplan–Meier survival analysis revealed a highly significant divergence in event-free survival favoring the SGLT2i group (Log-rank *P* = 0.005) (Figure 3). Cox regression confirmed that SGLT2i therapy was associated with a 38% relative risk reduction in the primary outcome (adjusted HR 0.62, 95% CI 0.44–0.87, *P* = 0.005) (Table 2). Subgroup analysis demonstrated consistent cardiovascular benefits across key strata, without significant interaction (*P* for interaction > 0.05) (Figure 4).

**Table 2:** Clinical efficacy and safety outcomes (post-PSM, intention-to-treat).

Outcomes	SGLT2i (*n* = 160)	Control (*n* = 160)	Adjusted HR (95% CI)	*P*-value
Primary Efficacy Endpoint	49 (30.6%)	73 (45.6%)	0.62 (0.44–0.87)	0.005
HHF	35 (21.9%)	52 (32.5%)	0.64 (0.43–0.95)	0.025
Cardiovascular Death	14 (8.8%)	21 (13.1%)	0.69 (0.40–1.18)	0.170
Primary safety endpoint (MAKE)	23 (14.4%)	27 (16.9%)	0.86 (0.52–1.43)	0.550
≥40% eGFR decline	17 (10.6%)	18 (11.3%)	–	0.850
Initiation of RRT	5 (3.1%)	8 (5.0%)	–	0.380
Renal Death	1 (0.6%)	1 (0.6%)	–	1.000
Secondary endpoints				
All-cause mortality	28 (17.5%)	34 (21.3%)	0.80 (0.49–1.30)	0.360
Severe AKI	15 (9.4%)	13 (8.1%)	1.16 (0.55–2.45)	0.690
Severe hyperkalemia	6 (3.8%)	11 (6.9%)	0.55 (0.21–1.48)	0.240

### Renal safety and eGFR trajectory

**Figure 5:**
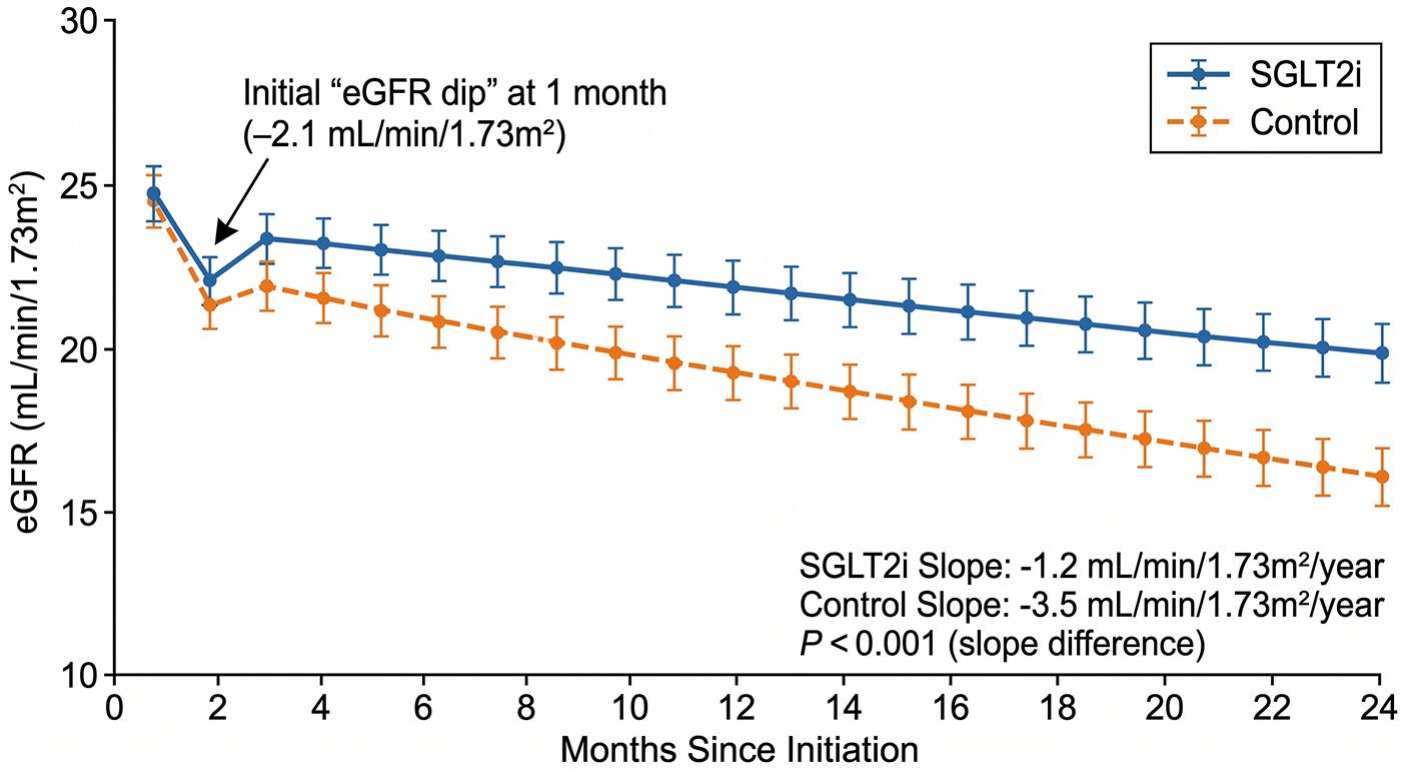
Long-term eGFR trajectory. Dynamic evolution of absolute eGFR values over 24 months. Error bars indicate 95% confidence intervals. Note the early initial dip followed by slope stabilization in the SGLT2i cohort.

Crucially, the primary safety outcome (MAKE) did not significantly differ between the SGLT2i and control groups (14.4% vs. 16.9%; adjusted HR 0.86, 95% CI 0.52–1.43, *P* = 0.55). The incidences of AKI and severe hyperkalemia were also comparable between the cohorts, with severe AKI occurring in 15 patients (9.4%) vs. 13 patients (8.1%) (*P* = 0.69). The linear mixed-effects model delineating the long-term eGFR trajectory revealed a characteristic pattern: the SGLT2i group experienced an initial “eGFR dip” at 1 month (−2.1 mL/min/1.73 m^2^), followed by a stabilization of renal function. Over 24 months, the annualized eGFR decline slope was significantly shallower in the SGLT2i group compared to the decline observed in the control group (−1.2 vs. −3.5 mL/min/1.73 m^2^ per year, *P* < 0.001) (Figure 5).

## Discussion

### Summary of principal findings

In this single-center, propensity score-matched, real-world retrospective cohort study, we investigated the long-term efficacy and renal safety of SGLT2 inhibitors in a highly vulnerable and under-studied population: patients with heart failure concomitant with advanced chronic kidney disease (CKD stage 4). Our principal findings suggest that even in this challenging clinical scenario with severely diminished renal reserve (median eGFR 22.4 mL/min/1.73 m^2^), SGLT2i therapy was consistently associated with favorable clinical outcomes. It was associated with a substantial 38% relative risk reduction in the primary composite endpoint of heart failure hospitalization or cardiovascular death. Importantly, our analysis suggests that this cardiovascular benefit does not appear to compromise renal safety. The incidence of major adverse kidney events, severe AKI, and hyperkalemia was statistically comparable to standard care, and SGLT2i utilization was associated with a significant attenuation of the long-term eGFR decline trajectory following an initial, predictable hemodynamic dip.

### Contextualization with existing literature

Our findings provide critical real-world validation to the extrapolated hypotheses derived from recent dedicated renal RCTs. The DAPA-CKD and EMPA-KIDNEY trials expanded the inclusion criteria to patients with eGFR down to 25 and 20 mL/min/1.73 m^2^, respectively (16, 20). However, the representation of patients with concurrent overt heart failure and stage 4 CKD in these trials was marginally small, leaving definitive conclusions for this specific intersection elusive. Furthermore, large HF trials (DAPA-HF, EMPEROR-Reduced) systematically excluded patients with eGFR < 30 mL/min/1.73 m^2^ (3, 21). Recent meta-analyses have attempted to pool subgroup data, suggesting maintained efficacy of SGLT2 inhibitors in lower eGFR strata, yet these were hindered by wide confidence intervals and the inherent limitations of *post-hoc* subgroup selection (22, 23). By employing rigorous propensity score matching to adjust for the immense confounding bias typical in severe renal impairment cohorts—where physicians often withhold life-saving therapies due to fear of acute kidney injury—our study robustly supports the hypothesis that SGLT2 inhibitors are safe and effective in advanced CKD. Our observed initial eGFR dip of approximately 2.1 mL/min/1.73 m^2^ aligns perfectly with the pharmacodynamic expectations described in less severe CKD cohorts (24), confirming that this mechanism is preserved even when functioning nephrons are scarce.

### Mechanistic insights

The sustained efficacy of SGLT2 inhibitors in patients with eGFR < 30 mL/min/1.73 m^2^ is biologically fascinating, given that the absolute glucosuric and diuretic effects of these agents are heavily dependent on the filtered load of glucose, which is drastically reduced in advanced CKD (25). The cardiovascular and nephroprotective benefits observed in this population strongly support the paradigm that SGLT2 inhibitors operate via pleiotropic, non-glycemic mechanisms (26). In the setting of severe nephron loss, the remaining glomeruli undergo profound hyperfiltration. SGLT2 inhibitors restore tubuloglomerular feedback by increasing sodium delivery to the macula densa, leading to afferent arteriolar vasoconstriction and a reduction in intraglomerular pressure (27). Furthermore, by shifting myocardial and renal metabolism towards ketone utilization, decreasing sympathetic nervous system overactivation, and alleviating renal cortical hypoxia, SGLT2 inhibitors mitigate the systemic inflammatory and profibrotic pathways that drive the cardiorenal vicious cycle (28).

### Clinical implications

The clinical implications of this study are immediate and actionable. The prevailing “prescription hesitation” or “renalism” regarding the initiation or continuation of SGLT2 inhibitors (SGLT2i) in patients experiencing worsening renal function down to CKD stage 4 deprives a high-risk population of a disease-modifying therapy (29). Our real-world data, in line with emerging evidence, advocate for a paradigm shift: an eGFR between 15 and 29 mL/min/1.73 m^2^ should not be viewed as an absolute contraindication or a reason for automatic withdrawal of SGLT2i in heart failure patients, as significant cardiorenal benefits are maintained even in advanced CKD (30, 31). Instead, clinicians should anticipate the early, reversible hemodynamic eGFR dip and focus on the long-term stabilization of renal function and the profound reduction in heart failure exacerbations, which are well-documented outcomes of SGLT2i therapy in this population (16).

### Limitations

Several limitations inherent to our study design must be acknowledged. First, as a retrospective observational study, despite the implementation of rigorous PSM, the potential for unmeasured residual confounding (such as dietary habits, frailty index, or precise volume status at baseline) cannot be entirely eliminated. Crucially, factors that typically dictate clinical hesitation—such as a history of recurrent urinary tract infections (UTIs), prior episodes of diabetic ketoacidosis (DKA), or severe peripheral arterial disease—were not fully captured in the PSM model and could independently influence both mortality and eGFR slopes. Second, this was a single-center study; thus, the findings may reflect specific institutional prescribing patterns and patient demographics, potentially limiting external validity. Third, continuous data on urinary albumin-to-creatinine ratio (UACR) were inconsistently documented in the EMR, precluding a robust longitudinal analysis of proteinuria dynamics. Finally, while the eGFR trajectory was analyzed over 24 months, the minimum follow-up for the latest enrolled patients was 12 months, which may be relatively short for definitively assessing long-term renal slopes. Previous meta-analyses have established that 3-year slopes possess a stronger association with hard renal endpoints (32). The relatively modest sample size, although adequately powered for the primary composite endpoint due to the high event rate in this frail population, warrants confirmation through larger, multicenter registries and ongoing dedicated RCTs.

## Conclusions

In conclusion, this propensity score-matched real-world study provides evidence suggesting that the utilization of SGLT2 inhibitors in patients with heart failure and advanced chronic kidney disease (stage 4) is associated with a significant reduction in cardiovascular death and heart failure hospitalizations. Furthermore, SGLT2i therapy is well-tolerated, is not associated with a significantly increased risk of severe acute kidney injury, and is associated with the preservation of long-term renal function trajectories. These findings support overcoming the clinical inertia surrounding SGLT2i prescription in the severe cardiorenal syndrome demographic.

## Data Availability

The original contributions presented in the study are included in the article/Supplementary Material, further inquiries can be directed to the corresponding authors.

## References

[1] Edwards NC Price AM Steeds RP Ferro CJ Townend JN. Management of heart failure in patients with kidney disease-updates from the 2021 ESC guidelines. Nephrol Dial Transplant. (2023) 38(8):1798–806. 10.1093/ndt/gfad011 36690349

[2] Cice G Calo L Monzo L. Sodium-glucose co-transporter 2 inhibitors for the treatment of cardio-renal syndrome. Eur Heart J Suppl. (2022) 24(Suppl I):I68–71. 10.1093/eurheartjsupp/suac101 36380781 PMC9653151

[3] McMurray JJV Solomon SD Inzucchi SE Køber L Kosiborod MN Martinez FA. Dapagliflozin in patients with heart failure and reduced ejection fraction. N Engl J Med. (2019) 381(21):1995–2008. 10.1056/NEJMoa1911303 31535829

[4] Packer M Anker SD Butler J Filippatos G Ferreira JP Pocock SJ. Influence of neprilysin inhibition on the efficacy and safety of empagliflozin in patients with chronic heart failure and a reduced ejection fraction: the EMPEROR-reduced trial. Eur Heart J. (2021) 42(6):671–80. 10.1093/eurheartj/ehaa968 33459776 PMC7878011

[5] Anker SD Butler J Filippatos G Ferreira JP Bocchi E Böhm M. Empagliflozin in heart failure with a preserved ejection fraction. N Engl J Med. (2021) 385(16):1451–61. 10.1056/NEJMoa2107038 34449189

[6] Perkovic V Jardine MJ Neal B Bompoint S Heerspink HJL Charytan DM. Canagliflozin and renal outcomes in type 2 diabetes and nephropathy. N Engl J Med. (2019) 380(24):2295–306. 10.1056/NEJMoa1811744 30990260

[7] Wanner C Inzucchi SE Zinman B. Empagliflozin and progression of kidney disease in type 2 diabetes. N Engl J Med. (2016) 375(18):1801–2. 10.1056/NEJMoa1515920 27806236

[8] Bakris G Oshima M Mahaffey KW Agarwal R Cannon CP Capuano G. Effects of canagliflozin in patients with baseline eGFR <30 ml/min per 1.73 m(2): subgroup analysis of the randomized CREDENCE trial. Clin J Am Soc Nephrol. (2020) 15(12):1705–14. 10.2215/CJN.10140620 33214158 PMC7769025

[9] Mottl AK Alicic R Argyropoulos C Brosius FC Mauer M Molitch M. KDOQI US commentary on the KDIGO 2020 clinical practice guideline for diabetes management in CKD. Am J Kidney Dis. (2022) 79(4):457–79. 10.1053/j.ajkd.2021.09.010 35144840 PMC9740752

[10] Jeong SJ Lee SE Shin DH Park IB Lee HS Kim KA. Barriers to initiating SGLT2 inhibitors in diabetic kidney disease: a real-world study. BMC Nephrol. (2021) 22(1):177. 10.1186/s12882-021-02381-3 33990175 PMC8122538

[11] Zhuo M Li J Buckley LF Tummalapalli SL Mount DB Steele DJR. Prescribing patterns of sodium-glucose cotransporter-2 inhibitors in patients with CKD: a cross-sectional registry analysis. Kidney360. (2022) 3(3):455–64. 10.34067/KID.0007862021 35582176 PMC9034822

[12] Ndebele P. The declaration of Helsinki, 50 years later. J Am Med Assoc. (2013) 310(20):2145–6. 10.1001/jama.2013.281316 24141794

[13] von Elm E Altman DG Egger M. The strengthening the reporting of observational studies in epidemiology (STROBE) statement: guidelines for reporting observational studies. J Clin Epidemiol. (2007) 60(6):344–9. 10.1016/j.jclinepi.2007.11.008 18313558

[14] Levey AS Eckardt KU Tsukamoto Y Levin A Coresh J Rossert J. Definition and classification of chronic kidney disease: a position statement from kidney disease: improving global outcomes (KDIGO). Kidney Int. (2005) 67(6):2089–100. 10.1111/j.1523-1755.2005.00365.x 15882252

[15] Hernán MA Wang W Leaf DE. Target trial emulation: a framework for causal inference from observational data. J Am Med Assoc. (2022) 328(24):2446–7. 10.1001/jama.2022.21383 36508210

[16] Heerspink HJL Stefánsson BV Correa-Rotter R Chertow GM Greene T Hou FF. Dapagliflozin in patients with chronic kidney disease. N Engl J Med. (2020) 383(15):1436–46. 10.1056/NEJMoa2024816 32970396

[17] Khwaja A. KDIGO clinical practice guidelines for acute kidney injury. Nephron Clin Pract. (2012) 120(4):c179–184. 10.1159/000339789 22890468

[18] White IR Royston P Wood AM. Multiple imputation using chained equations: issues and guidance for practice. Stat Med. (2011) 30(4):377–99. 10.1002/sim.4067 21225900

[19] Wang Y Cai H Li C Jiang Z Wang L Song J. Optimal caliper width for propensity score matching of three treatment groups: a Monte Carlo study. PLoS One. (2013) 8(12):e81045. 10.1371/journal.pone.0081045 24349029 PMC3859481

[20] Herrington WG Staplin N Wanner C Green JB Hauske SJ Emberson JR. Empagliflozin in patients with chronic kidney disease. N Engl J Med. (2023) 388(2):117–27. 10.1056/NEJMoa2204233 36331190 PMC7614055

[21] Beldhuis IE Martens P Ter Maaten JM. Early changes in renal function after sodium-glucose cotransporter 2 inhibitor initiation in EMPEROR-reduced: the end of the dilemma? Eur J Heart Fail. (2022) 24(10):1840–3. 10.1002/ejhf.2659 35999649

[22] Mavrakanas TA Tsoukas MA Brophy JM Sharma A Gariani K. SGLT-2 inhibitors improve cardiovascular and renal outcomes in patients with CKD: a systematic review and meta-analysis. Sci Rep. (2023) 13(1):15922. 10.1038/s41598-023-42989-z 37741858 PMC10517929

[23] Vaduganathan M Docherty KF Claggett BL Jhund PS de Boer RA Hernandez AF. SGLT-2 inhibitors in patients with heart failure: a comprehensive meta-analysis of five randomised controlled trials. Lancet. (2022) 400(10354):757–67. 10.1016/S0140-6736(22)01429-5 36041474

[24] McMurray JJV Wheeler DC Stefánsson BV Jongs N Postmus D Correa-Rotter R. Effects of dapagliflozin in patients with kidney disease, with and without heart failure. JACC Heart Fail. (2021) 9(11):807–20. 10.1016/j.jchf.2021.06.017 34446370

[25] Li N Lv D Zhu X Wei P Gui Y Liu S. Effects of SGLT2 inhibitors on renal outcomes in patients with chronic kidney disease: a meta-analysis. Front Med. (2021) 8:728089. 10.3389/fmed.2021.728089 PMC8591237 34790672

[26] Preda A Montecucco F Carbone F Camici GG Lüscher TF Kraler S. SGLT2 inhibitors: from glucose-lowering to cardiovascular benefits. Cardiovasc Res. (2024) 120(5):443–60. 10.1093/cvr/cvae047 38456601 PMC12001887

[27] Wonnacott A. The emerging pillars of chronic kidney disease: no longer a bystander in metabolic medicine. Clin Med. (2023) 23(3):254–8. 10.7861/clinmed.2023-RM1 PMC11046543 37236791

[28] Girardi ACC Polidoro JZ Castro PC Pio-Abreu A Noronha IL Drager LF. Mechanisms of heart failure and chronic kidney disease protection by SGLT2 inhibitors in nondiabetic conditions. Am J Physiol Cell Physiol. (2024) 327(3):C525–44. 10.1152/ajpcell.00143.2024 38881421

[29] Thomas MC Neuen BL Twigg SM Cooper ME Badve SV. SGLT2 inhibitors for patients with type 2 diabetes and CKD: a narrative review. Endocr Connect. (2023) 12(8):e230005. 10.1530/EC-23-0005 37159343 PMC10448577

[30] Li N Zhou G Zheng Y Lv D Zhu X Wei P. Effects of SGLT2 inhibitors on cardiovascular outcomes in patients with stage 3/4 CKD: a meta-analysis. PLoS One. (2022) 17(1):e0261986. 10.1371/journal.pone.0261986 35020750 PMC8754287

[31] Roddick AJ Wonnacott A Webb D Watt A Watson MA Staplin N. UK Kidney association clinical practice guideline: sodium-glucose co-transporter-2 (SGLT-2) inhibition in adults with kidney disease 2023 UPDATE. BMC Nephrol. (2023) 24(1):310. 10.1186/s12882-023-03339-3 37880609 PMC10598949

[32] Inker LA Heerspink HJL Tighiouart H Levey AS Coresh J Gansevoort RT. GFR slope as a surrogate end point for kidney disease progression in clinical trials: a meta-analysis of treatment effects of randomized controlled trials. J Am Soc Nephrol. (2019) 30(9):1735–45. 10.1681/ASN.2019010007 31292197 PMC6727261

